# Innovate to heal: 3D bioprinting tackles epidermolysis bullosa

**DOI:** 10.1097/MS9.0000000000004150

**Published:** 2025-10-17

**Authors:** Kinza Tanveer, Muhammad Talha, Araj Naveed Siddiqui, Hermann Yokolo

**Affiliations:** a Department of Medicine, Karachi Metropolitan University, Karachi, Pakistan; b King Edward Medical University, Lahore, Pakistan; c Department of Medicine, Jinnah Sindh Medical University, Karachi, Pakistan; d Department of Research, Medical Research Circle (MedReC), Goma, DR Congo

*Dear editor,*

Epidermolysis bullosa (EB) is a rare inherited condition that causes the skin and mucous membranes to be extremely fragile, leading to painful blisters and wounds from even minor friction or pressure. It is caused by mutations in genes that affect how the skin layers attach. The condition weakens the bond between the epidermis and dermis, causing fragile skin that blisters easily. It can be inherited dominantly or recessively. There are four main types: EB simplex, junctional EB, dystrophic EB, and Kindler syndrome. The severity of EB varies from mild to life-threatening. In some types and subtypes, serious complications can affect the eyes, ears, nose, upper airways, digestive system, and urinary tract^[1]^. The objective of this article is to highlight the innovative therapeutic potential of three-dimensional (3D) bioprinting in the treatment of EB, highlighting the scientific advances, the expected clinical benefits, and the need for equitable access to this emerging technology through appropriate health policies. This article complies with the TITAN 2025 guidelines – repressing the reporting and use of artificial intelligence (AI)^[2]^.

3D bioprinting is an emerging technology with the potential to transform the treatment of skin wounds caused by burns, ulcers, and genetic skin disorders. It is being explored to create human skin replacements that restore function and support healing after injury^[3]^. 3D bioprinting is a technique that uses additive manufacturing to layer biomaterials and living cells, creating biological constructs that replicate the structure and function of natural tissues and organs. This process deposits biocompatible materials layer by layer onto substrates to build complex, functional tissues^[4]^. The different types of 3D bio printers are found in Table 1

**Table 1:** Comparison of the main types of bioprinters for skin applications

Type of bioprinter	Operating principle	Main applications	Benefits	Boundaries	References
Extrusion-based	Continuous deposition of bio-ink via a pressurized nozzle	Skin grafts, soft tissues, wound models	Compatible with viscous bio-inks, construction of bulky tissues	Lower resolution, mechanical stress on cells	^[5,6,7]^
Inkjet (drop-on-demand)	Depositing individual drops of bio-ink	Micro-tissues, cellular models, *in vitro* tests	High resolution, low cost, good cell viability	Limited to low viscosity bio-inks, low printing speed	^[5,6,7]^
Laser-assisted (LIFT)	Bioink transfer via laser energy	Micro-patterning, delicate fabrics	Very high resolution, no nozzle so no clogging	Expensive equipment, limited production	^[5,6,7]^
Stereolithography (SLA)/digital light processing (DLP)	Photopolymerization of photosensitive resins or bio-inks	Structural models, graft support	High precision, complex structures	Limited to photosensitive materials, possible toxicity of photoinitiators	^[5,6,7]^

There are two primary methods of 3D bioprinting, in situ and *in vitro*; both are currently under study for their applications in skin replication and healing wounds (Table 2). 3D bioprinting offers promising potential for wound healing in patients with EB, particularly recessive dystrophic epidermolysis bullosa, by enabling structured skin reconstruction. The printed grafts incorporate genetically modified cells or therapeutic molecules, facilitating skin regeneration and restoring damaged tissue function. This approach could strengthen impaired dermo-epidermal adhesions and locally deliver reparative agents, thus contributing to targeted wound repair. However, although the technology shows potential benefits, the precise mechanisms by which these grafts interact with the skin microenvironment and enable healing remain unclear^[5]^.

**Table 2:** Comparison of in situ and *in vitro* 3D bioprinting methods for skin regeneration

Bioprinting method	Description	Benefits	Boundaries	References
*In situ*	The skin is printed directly onto the patient’s wound, layer by layer.	- Direct adaptation to wound topography	- Technical challenges related to precision on irregular surfaces	^[6,7]^
		- Reduced time between printing and implantation		
			- Difficulty in maintaining cell viability in clinical conditions	
*In vitro*	Skin grafts are printed on a support in the laboratory and then transferred to the wound.	- Precise control of structure and composition	- Requires transfer and implantation, with risks of deformation or rejection	^[6,7]^
		- Possibility of maturation and prior testing		
			- Longer and more expensive	

Collagen-based biomaterials are widely used as 3D substrates for cell culture and are considered promising scaffolds for engineering artificial tissues. These include type I collagen of bovine or porcine origin, as well as recombinant human collagen. One of the greatest strengths of 3D bioprinted materials incorporating collagen is their high potential for future clinical success. This is largely because collagen-based biomaterials have already been extensively and successfully used in clinical settings for many years. Collagen’s natural biocompatibility and low risk of immune rejection make it an ideal scaffold material. Therefore, to maintain this low immunogenicity in 3D bioprinted constructs, it is crucial to use highly purified collagen solutions that are free from any immunogenic impurities or contaminants^[6]^.

There is no doubt that 3D bioprinting technology holds great potential to produce highly precise, reproducible, and fully functional synthetic tissues and organs. However, a major challenge that remains is scaling up the manufacturing of 3D-printed skin to treat large tissue defects and serve many patients. Scaling up involves difficulties such as maintaining high resolution while printing large volumes and addressing storage challenges, since the skin substitutes are living tissues^[5]^. Optimizing bioinks is essential to ensure reliable printability, reproducibility, and appropriate spatial organization, which remains complex. Furthermore, the translation of *in vitro* results to *in vivo* conditions is hampered by a lack of knowledge of the molecular signals that effectively activate healing. Immunological issues constitute another major obstacle. The use of autologous cells, such as induced Pluripotent Stem Cells, is not always feasible, particularly in cases of genetic diseases. Allogeneic cell banks, for their part, face challenges due to the diversity of Human Leukocyte Antigen (HLA) haplotypes, requiring immunomodulatory approaches to limit rejection. Finally, clinical translation requires strict control of cell quality and the conduct of rigorous clinical trials, where standard comparators such as autograft remain difficult to surpass^[3,7]^.

In conclusion, 3D bioprinting appears to be an innovative and promising avenue for the treatment of EB, offering the possibility of generating functional and biocompatible skin substitutes capable of restoring the skin barrier and improving patients’ quality of life. However, its clinical integration still faces several limitations, including bioink optimization, tissue reproducibility, immunological compatibility, and the difficulty of translating *in vitro* results into *in vivo* conditions. In the future, the development of smarter bioinks, integrating molecular healing signals, and the use of genetically corrected autologous cells could overcome some of these obstacles. In parallel, the establishment of standardized protocols, the implementation of scalable production platforms, and support by health policies promoting equity of access will be essential to transform this emerging technology into a safe, effective, and accessible therapeutic solution for EB patients.

## Data Availability

Not applicable.
